# Collaboration Between Emergency Medicine and Radiology in Regional Hospitals Improves Initial Diagnosis Accuracy: A Descriptive Study With Case Series

**DOI:** 10.7759/cureus.79940

**Published:** 2025-03-03

**Authors:** Natsuyo Shinohara, Shunsuke Nakamura, Takumu Maruyama, Ryo Ohno, Osamu Honda

**Affiliations:** 1 Department of Emergency Medicine, Okayama Rosai Hospital, Okayama, JPN; 2 Department of Radiology, Okayama Rosai Hospital, Okayama, JPN

**Keywords:** collaboration, diagnostic imaging, emergency medicine, initial diagnosis, radiologist

## Abstract

Imaging plays a critical role in emergency medicine, and effective communication between radiologists and emergency physicians is essential for accurate diagnoses. Timely and accurate interpretation of imaging is particularly vital in critical cases. This case series highlights three emergency cases that emphasize the importance of timely imaging and interdepartmental collaboration. Case 1 is a 63-year-old woman with diabetic ketoacidosis and shock, where imaging revealed pancreatitis and hemoperitoneum. Case 2 is a 72-year-old male patient diagnosed with strangulated ileus via CT, leading to prompt surgical intervention. Case 3 is a 92-year-old female patient who had multiple problems, where imaging revealed pulmonary embolism, cerebral infarction, and suspected appendiceal cancer. Effective communication between emergency physicians and radiologists is paramount for enabling rapid and accurate diagnoses, ultimately improving patient care. Strengthening this collaboration reduces stress among healthcare professionals and enhances emergency care, particularly in resource-limited hospitals.

## Introduction

Imaging has become an integral component of emergency medicine, particularly in contemporary Japan, where the aging population has heightened its importance in patient management and outcomes [1,2]. Its impact on diagnosis and patient outcomes is particularly significant in the elderly population, where vague symptoms and a high rate of imaging referrals are common.

For instance, at a secondary emergency hospital in Japan, such as our own, the implementation rate of imaging-based diagnosis exceeds 50%. A report indicates that the missed diagnosis rate on holidays and at night―when staff shortages and the absence of radiology specialists pose challenges―is 1.1% [3]. 

In emergency medicine, prompt and precise interpretation is paramount, as accurate imaging assessments enable swift diagnosis and treatment―an especially critical factor in life-threatening situations [4]. The efficacy of this collaborative approach hinges effective direct "face to face" communication between emergency physicians and radiologists.

This study aims to evaluate the impact of real-time collaboration between emergency physicians and radiologists on diagnostic accuracy and patient outcomes in regional hospitals. At our hospital, the emergency and radiology departments engage in daily communication. We have documented multiple cases in which prompt and accurate diagnoses were achieved. We assert that such interdepartmental collaboration is imperative for local emergency hospitals struggling with chronic staff shortages, and we present it here as one of our proposed countermeasures.

## Case presentation

This case series presents three emergency department (ED) cases involving various acute conditions. A detailed discussion is provided on the clinical presentation, diagnostic approach, management, and patient outcomes, with the objective of highlighting critical decision-making in emergency medicine.

Case 1: diabetic ketoacidosis and shock due to facial cellulitis

**Figure 1:**
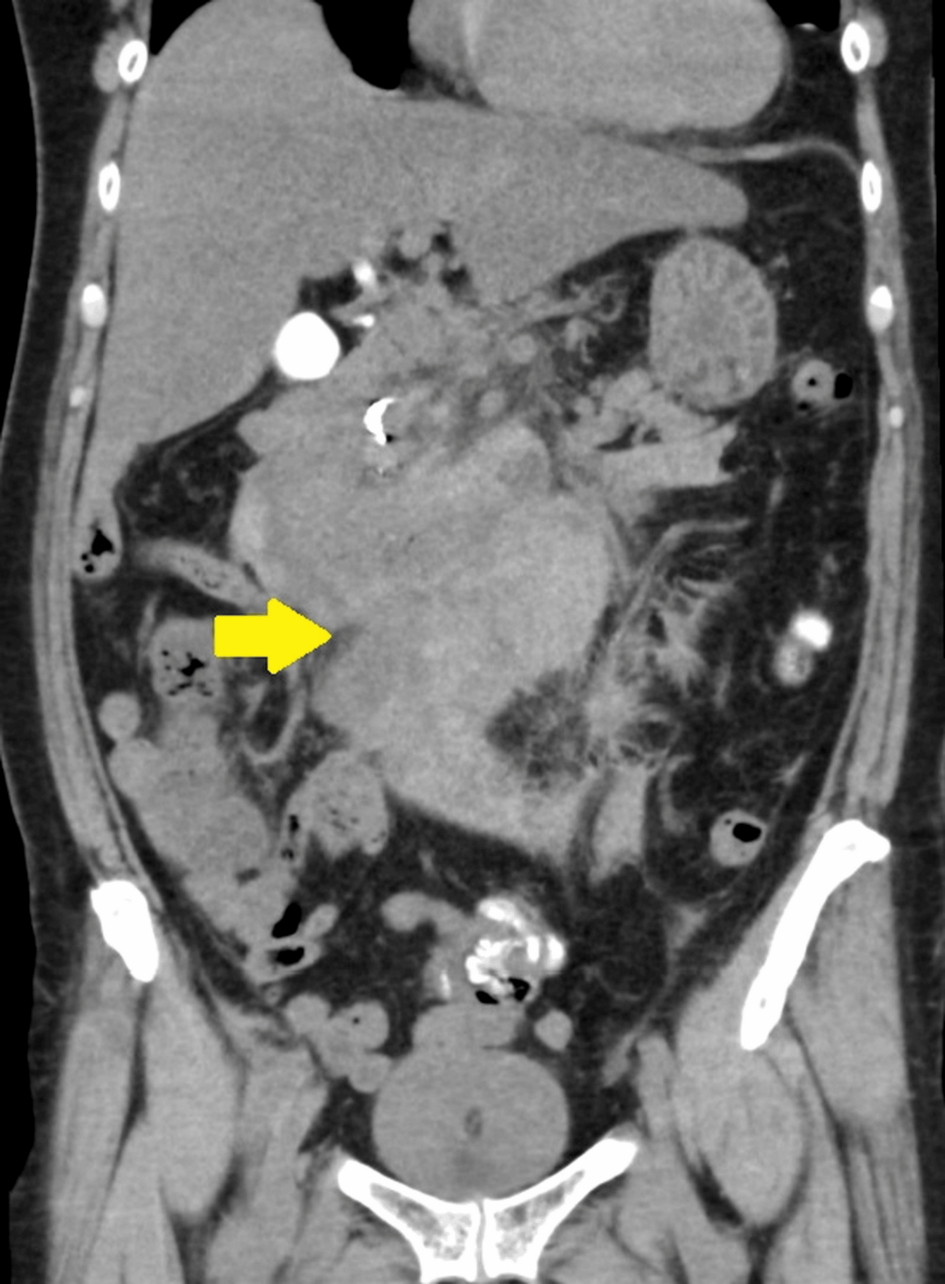
Ruptured pancreaticoduodenal artery aneurysm due to arch-ligament compression syndrome Subsequent computed tomography (CT) imaging revealed inflammation extending from the duodenum to the pancreas with hemoperitoneum, raising concerns for acute pancreatitis or pseudoaneurysm rupture (the yellow arrow).

A 63-year-old female patient was admitted to the hospital with complaints of dyspnea, profuse sweating, and right facial cellulitis with an ear laceration. Her medical history included untreated impaired glucose tolerance. On presentation, the patient exhibited blood pressure (BP) of 60/42 mmHg, heart rate (HR) of 97 beats per minute (bpm), and oxygen saturation (SpO_2_) of 99% (on six liters of oxygen via mask). Arterial blood gas analysis revealed metabolic acidosis with hyperglycemia, leading to a suspicion of diabetic ketoacidosis (DKA) with hypovolemic shock. Fluid resuscitation was promptly initiated, resulting in a swift hemodynamic response. Subsequent computed tomography (CT) imaging revealed inflammation extending from the duodenum to the pancreas with hemoperitoneum, raising concerns about acute pancreatitis or pseudoaneurysm rupture (Fig. 1). Given the potential need for interventional radiology, the patient was transferred to an advanced medical care institution for further management.

Case 2: strangulated ileus requiring emergency surgery

**Figure 2:**
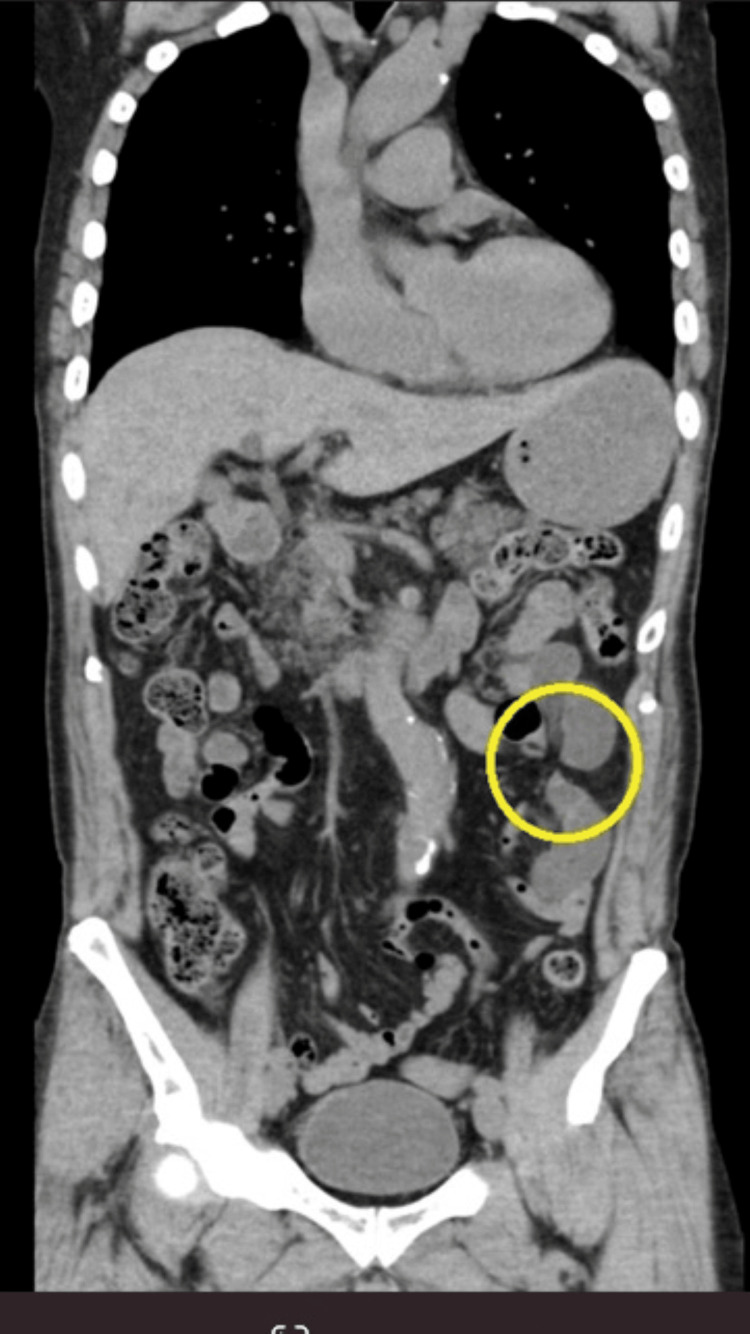
Strangulated ileus (closed loop) seen on plain CT The patient was administered intravenous acetaminophen for pain management, and non-contrast CT imaging suggested a strangulated ileus (the yellow circle).

A 72-year-old male patient presented with severe lower abdominal pain and vomiting. Due to the severity of the pain, a consultation with the ED was requested. On arrival, vital signs revealed a Glasgow Coma Scale (GCS) of E3V4M6, with BP of 130/62 mmHg, HR of 73 bpm, and RR of 41 breaths per minute. The patient is in a state of restlessness or agitation because a physical examination revealed severe lower abdominal pain, hyperventilation due to pain, intermittent waves of pain, and tenderness localized to the lower mid-abdomen without peritoneal signs. Arterial blood gas analysis revealed elevated lactate levels (8.1 mmol/L). Non-contrast CT imaging suggested a strangulated ileus because his kidney function was unknown at that time (Fig. 2), which was later confirmed by contrast-enhanced CT. The gastrointestinal surgery team was consulted, and emergency surgical intervention was performed.

Case 3: pulmonary embolism and cerebral infarction with suspected cecal infiltration from appendiceal cancer

**Figure 3:**
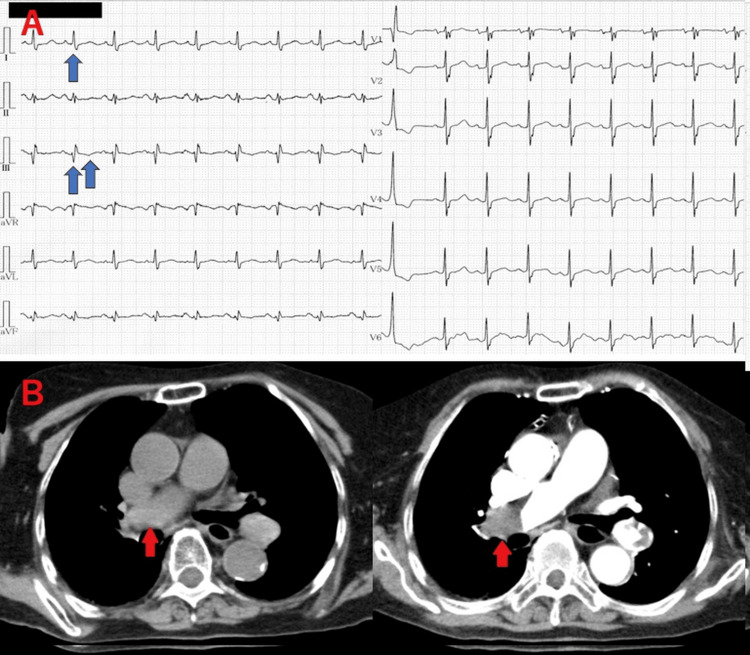
Pulmonary thromboembolism suspected by electrocardiogram and diagnosed by plain CT Electrocardiogram (ECG) showed S1Q3T3 pattern (A, blue arrows) and computed tomography (CT) findings were suggestive of pulmonary embolism (B, red arrows).

A 92-year-old female patient was found to be in a state of altered consciousness. She had vomited after consuming watermelon, prompting an emergency call. On arrival, the patient's vital signs were as follows: GCS E3V3M6, BP 106/81 mmHg, HR 106 bpm, SpO_2_ 72% at room air and elevated to 96% on 10-liter oxygen by non-rebreather mask, and RR 30 breaths per minute. Physical examination revealed altered consciousness, hypoxemia, and impaired speech assessment due to pre-existing hearing loss. The electrocardiogram (ECG) showed an S1Q3T3 pattern (Fig. 3A), and CT findings suggested pulmonary embolism (PE) (Fig. 3B). The fact that the disturbance of consciousness is prolonged and D-dimer levels were significantly elevated prompted further imaging, including a brain MRI and contrast-enhanced CT. Imaging confirmed the presence of PE, cerebral infarction, and suspected appendiceal cancer with cecal infiltration. Consequently, the cardiology, neurosurgery, and gastrointestinal surgery teams were consulted. Treatment priorities were established for the patient's pulmonary embolism and stroke, and she was admitted to the intensive care unit (ICU) for further management.

## Discussion

Imaging diagnosis is invaluable in determining a patient’s final diagnosis and clinical outcome. It is encouraging to see an increasing utilization of imaging, especially among elderly patients, who often present vague symptoms. a patient’s final diagnosis and clinical outcome. It is encouraging to see an increasing utilization of imaging, especially among elderly patients, who often present vague symptoms.

One study reported that 99.0% of X-rays interpreted by attending physicians during initial ED evaluations were correctly assessed [5]. These findings suggest that emergency physicians are capable of accurate interpretation, although their accuracy may not reach the levels of radiologists (94-97%) [6]. However, the rate of agreement between emergency physicians and radiologists was 84.3% for normal cases but lower for cases of congestive heart failure (41.4%) and pneumonia (41.4%). While ED physicians may occasionally fail to detect radiographic abnormalities, there is always room for improvement. The rate of agreement is lower for diagnostic categories such as pneumonia and congestive heart failure [5]. Timely and precise assessments are key to the swift diagnosis and treatment of patients, particularly in life-threatening situations. When emergency physicians and radiologists collaborate effectively, they can achieve faster and more accurate diagnoses. However, environmental, personnel, and institutional factors can sometimes hinder communication. Nevertheless, fostering strong interdisciplinary cooperation can overcome these barriers and significantly improve patient outcomes. Personal and institutional factors can sometimes hinder communication. Nevertheless, fostering strong interdisciplinary cooperation can overcome these barriers and significantly improve patient outcomes.

At our hospital, the emergency and radiology departments engage in daily communication, and we have documented multiple cases where accurate diagnoses were achieved promptly. We believe that such interdepartmental collaboration is essential for community emergency hospitals struggling with chronic staff shortages, and we are pleased to share our approach as a model. The case studies presented here exemplify how close collaboration between emergency physicians and radiologists facilitates rapid and accurate diagnoses. At our hospital, fostering daily trust between the emergency and radiology departments has helped bridge communication gaps. For example, imaging test orders from the ED include detailed clinical history, physical examination findings, differential diagnoses under consideration, and the purpose of the imaging study. This approach benefits not only emergency physicians but also radiologists, reducing their cognitive load and clarifying key areas of focus. We have observed multiple instances where open access to the reading room and daily interactions between emergency physicians and radiologists led to more efficient diagnoses. This case series underscores the complexity of ED cases and the necessity of rapid and accurate clinical decision-making. For instance, in the case of DKA and shock, the identification of underlying pancreatitis emphasized the importance of further imaging. Due to the swift and effective communication between the emergency physician and the radiologist, it took only 67 minutes from the patient's arrival to undergoing a CT scan. In addition, the radiologist's report was reviewed within 43 minutes, and the diagnosis was established based on imaging findings alone. Similarly, the case of strangulated ileus demonstrated the benefits of early recognition and prompt intervention. The emergency physician, who suspected strangulated ileus early on, immediately consulted with the radiologist in the imaging room, leading to a definitive diagnosis just 64 minutes after the imaging examination began. The cases of pulmonary embolism and cerebral infarction highlight the challenges of managing multiple organ failures in elderly patients, necessitating a multidisciplinary approach. 

In one case, an experienced emergency physician suspected pulmonary embolism based on ECG findings and promptly consulted the radiologist. As a result, a diagnosis was made within 30 minutes of the patient's arrival using a non-contrast CT scan alone. The emergency physician will often proceed to the radiology suite to inquire about the findings, or alternatively, the radiologist will attend the emergency room to convey the results directly, and we have documented multiple cases where accurate diagnoses were achieved promptly. We believe that such interdepartmental collaboration is essential for community emergency hospitals struggling with chronic staff shortages, and we are pleased to share our approach as a model. The case studies presented here exemplify how close collaboration between emergency physicians and radiologists facilitates rapid and accurate diagnoses. At our hospital, fostering daily trust between the emergency and radiology departments has helped bridge communication gaps. For example, imaging test orders from the emergency department include detailed clinical history, physical examination findings, differential diagnoses under consideration, and the purpose of the imaging study. This approach benefits not only emergency physicians but also radiologists, reducing their cognitive load and clarifying key areas of focus. We have observed multiple instances where open access to the reading room and daily interactions between emergency physicians and radiologists led to more efficient diagnoses. This case series underscores the complexity of emergency department cases and the necessity of rapid and accurate clinical decision-making. For instance, in the case of DKA and shock, the identification of underlying pancreatitis emphasized the importance of further imaging. Due to the swift and effective communication between the emergency physician and the radiologist, it took only 67 minutes from the patient's arrival to undergoing a CT scan. Additionally, the radiologist's report was reviewed within 43 minutes, and the diagnosis was established based on imaging findings alone. Similarly, the case of strangulated ileus demonstrated the benefits of early recognition and prompt intervention. The emergency physician, who suspected strangulated ileus early on, immediately consulted with the radiologist in the imaging room, leading to a definitive diagnosis just 64 minutes after the imaging examination began. The cases of pulmonary embolism and cerebral infarction highlight the challenges of managing multiple organ failures in elderly patients, necessitating a multidisciplinary approach. In one case, an experienced emergency physician suspected pulmonary embolism based on ECG findings and promptly consulted the radiologist. As a result, a strong suspicion of PE could be made within 30 minutes of the patient's arrival using a non-contrast CT scan alone.

However, several limitations should be acknowledged. At our facility, we are fortunate to have an adequate number of emergency physicians and radiologists who can discuss imaging findings in real time. Initial emergency room evaluations are conducted by emergency medicine specialists, but in many community hospitals, they are performed by physicians from other specialties or by residents without specialized training in emergency medicine.

## Conclusions

This case series highlights the critical role of imaging in emergency medicine and the importance of effective collaboration between emergency physicians and radiologists. Efficient interdepartmental communication facilitated rapid and accurate diagnoses, ultimately improving patient outcomes. The collaborative efforts of the team played a key role in minimizing delays in diagnostic imaging. These findings emphasize the importance of fostering a cooperative environment, streamlining the diagnostic process, and enhancing interdisciplinary communication to optimize initial treatment and ensure patient safety in regional emergency medicine.
